# Primary spontaneous pneumothorax in a term neonate

**DOI:** 10.4314/ahs.v22i4.21

**Published:** 2022-12

**Authors:** Abiola O Adekoya, Adesola O Adekoya, Lukmon O Amosu, Ayodeji A Olatunji, Mojisola A Olusola-Bello, Olubukola O Ashaolu

**Affiliations:** 1 Department of Radiology, Olabisi Onabanjo University Teaching Hospital, Sagamu, Nigeria; 2 Department of Pediatrics, Babcock University Teaching Hospital, Ilisan, Nigeria; 3 Department of Surgery, Olabisi Onabanjo University Teaching Hospital, Sagamu, Nigeria

## Abstract

Pneumothorax is a rare but recognized cause of respiratory distress in the newborn. It can occur spontaneously or post-traumatic. We report our experience in a term male neonate who had primary spontaneous pneumothorax. He had no surgical intervention but completely recovered with conservative management and supplemental oxygen.

## Introduction

Pneumothorax (PTX) is the presence of air in the potential space between the parietal and visceral pleurae of the thoracic cavity, with consequent increase in the intrapulmonary space pressure exceeding that of the extra pleural pressure.^1^ It is not a disease in itself, but may be life-threatening. The incidence of PTX is as low as 1% in term neonates and as high as 6–10% in preterm and very low birth weight (VLBW) babies, likely secondary to poor lung compliance.^2^ Pneumothorax can occur spontaneously or secondary to trauma.^3^ Trauma-related PTX can be iatrogenic or accidental. Spontaneous pneumothorax (SP) can be primary (without clinical or radiographical apparent lung or chest wall disease) or secondary (complication of chronic or acute lung disease and use of mechanical ventilation).^3^ Known risk factors contributing to neonatal pneumothorax are male gender, low birth weight, prematurity, post-maturity, use of mechanical ventilation and Caesarean-section in term infants.^4^ Pneumothorax is a recognized cause of respiratory distress in healthy neonates and may occur in the absence of risk factor at birth.^5^ Its early diagnosis and prompt treatment are important and could be life-saving. In this case report, the clinical presentation, chest radiographs, treatment, and radiologic follow-up process of a healthy term newborn with primary spontaneous pneumothorax are shared. This will prompt neonatal care-providers to consider SP, in the evaluation of newborns with sudden and worsening postpartum respiratory distress, and oxygen deterioration requiring respiratory support.

## Case report

A 3.2kg male neonate, delivered at 38 weeks gestational age via spontaneous vaginal delivery in our facility suddenly developed respiratory distress post-delivery. Mother attended ante-natal clinic regularly and had two obstetric scans which showed no fetal abnormality. There was no history of maternal diabetes or hypertension. Labour was spontaneous, not augmented and lasted for about 6 hours. Apgar scores at birth were 8 and 10 at 1st and 5th minutes respectively. No active intrapartum resuscitation was required. There was no history of trauma during delivery, the amniotic fluid was not stained with meconium and the placenta was delivered intact. About an hour after birth, he suddenly developed difficulty in breathing and was admitted into the neonatal emergency unit.

**Figure 1:**
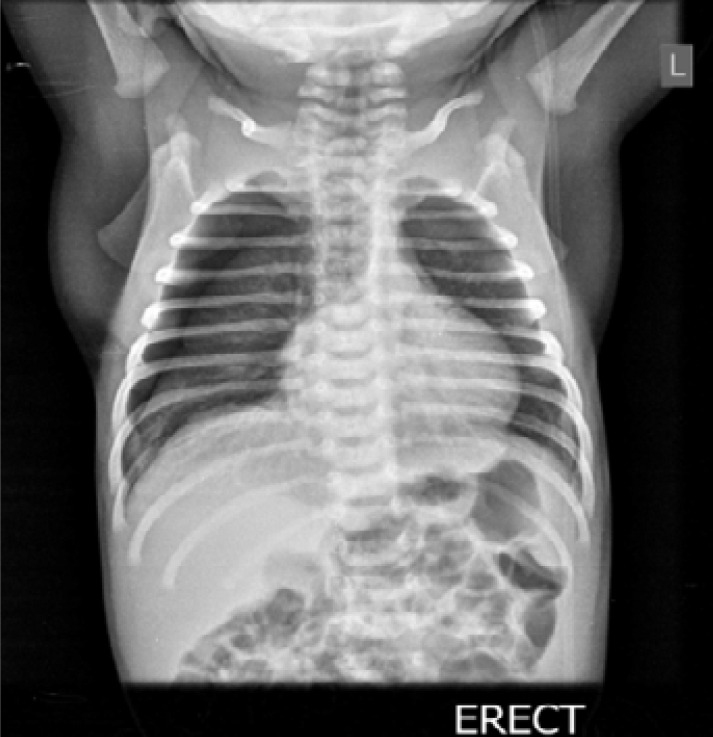
Chest X-ray 1st day of life showed right pneumothorax with lung collapse.

On physical examination, he was tachypneic with a respiratory rate of 86 cycles per minute, tachycardiac with heart rate of 178 beats per minute. The blood pressure was 64/42mmHg. There were marked intercostal withdrawal, reduced breath sounds with hyper-resonant percussion notes in the right hemithorax. The heart sounds were normal and there was no murmur. The abdomen was soft with no palpable organomegaly. Arterial oxygen saturation (SpO2) via pulse oximeter was 88%. He was commenced on 100% oxygen via nasal prong at 4litres per minute and oxygen saturation improved to 95%. A clinical diagnosis of right-sided pneumothorax was entertained. An urgent chest radiograph (CXR) in erect view showed hyperlucency devoid of lung markings in the right hemithorax, deepened right hemi-diaphragmatic sulcus and a medially placed wedge-shaped, soft tissue mass, consistent with right lung collapse. The right hemidiaphragm and ribs were not flattened. The left hemithorax and cardiac silhouette were normal (Figure 1). A diagnosis of right-sided primary pneumothorax with partial right lung collapse was made.

**Figure 2:**
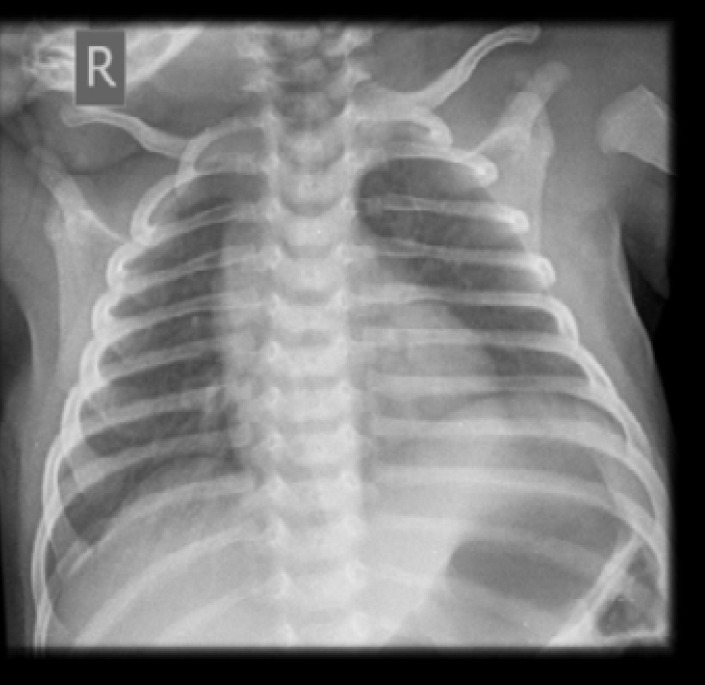
Chest X-ray on 10th day of life showed total resolution of pneumothorax and re-expansion of the right lung.

The baby had full blood work-up for sepsis which were negative. Serum electrolytes, urea and blood glucose were within normal limits. Echocardiography and abdominal ultrasonography were normal. He was managed on high-flow supplemental oxygen via nasal prongs and placed on intravenous fluid (10% dextrose water). After 24 hours of oxygen therapy, grunting subsided and there was marked improvement in respiration although, mild subcostal recession was still present. Respiratory and heart rates reduced to 60 cycles per minute and 136 beats per minute respectively. He was able to maintain 95% to 98% oxygen saturation on 100% oxygen via nasal catheter at 2litres per minute. A repeat CXR after 24 hours showed no significant change in the volume of the pneumothorax. Supplemental oxygen therapy was continued. By the 4th day of life, the baby was no longer breathless and vital signs had normalized. Nasogastric tube was introduced for graded feeding of expressed breast milk, which was well tolerated, and he had good bowel movements. Serial chest radiographs taken over days showed progressive reduction in the volume of the pneumothorax, re-expansion of the affected lung and overall clinical improvement. Nasogastric tube was removed on the 8^th^ day of life. Patient tolerated and did well on direct breast feeding with no vomiting or aspiration and there was no recurrent episode of respiratory distress. A CXR taken on 10th day of life showed total resolution of the right pneumothorax with significant re-expansion of the collapsed right lung (Figure 2).

He was discharged home on the 14^th^ day of life. He did not require any surgical intervention in form of chest tube placement or needle thoracentesis. At follow up visits, there were no remarkable chest findings and no repeat episode of respiratory distress. He was discharged from the clinic after one year.

## Discussion

The pathogenesis of primary SP in the newborn is uncertain. However, it results from alveoli rupture either secondary to high pressure needed to expand previously uninflated lungs or from uneven distribution of inflating pressures among the alveoli.^6^ Neonatal SP is commoner in premature, postmature and male infants.^5^ It is mostly unilateral and commoner on the right side of the lung.^6^ Our patient was term male neonate that required no intrapartum resuscitation but developed SP in his right lung at the 1^st^ day of life. He had no underlying lung pathology and no known risk factor aside male gender.

Chest radiograph (CXR) is usually the first investigation tool in the assessment of neonatal pneumothorax than the gold standard chest computed tomography (CT) which is usually not technically feasible with patient's exposure to ionizing radiation. It is simple, fast, non-invasive, inexpensive, relatively available and equally useful and safe for serial monitoring as treatment progresses. However, it is less sensitive than chest CT in the detection of small pneumothorax, bullae and blebs as significant amount of air must accumulate in the pleural space before it can be confidently recognized. Alternatively, bedside lung ultrasound (LUS) is gaining momentum in the diagnosis of PTX in newborns with a reportedly higher sensitivity and specificity than the CXR. It is reproducible, does not use ionizing radiation, and able to confirm the level of lung point for thoracocentesis. However, it is operator dependent and requires quality assurance.^7^ It is not yet incorporated in the diagnostic work-up of neonatal PTX as seen in the adults.

There is no consensus guideline for the management of neonatal PTX as available for adults. Treatment decisions are based on the severity of respiratory distress and the development of complications such as primary pulmonary hypertension during the course of illness rather than the size of the pneumothorax.^8^ Conservative management via observation and oxygen therapy with or without use of mechanical ventilation to support breathing may therefore suffice for patients with mild to moderate respiratory distress. It is a safe and efficacious treatment alternative to surgical interventional management. Reportedly, it has a lower PTX recurrence, lower risk of adverse events such as haemorrhage, parenchymal laceration, hypotension, and damage to deeper structures; albeit with longer hospital stay as observed in this reported case.^9^ Conservative treatment was reportedly successful in the care of bilateral SP in a newborn with cystic fibrosis with complete PTX resolution.^10^ In cases of persistence or reoccurrence of PTX, tension PTX with severe lung collapse, development of complications, worsening and distressing symptoms, or patients at special risk, tube thoracostomy with small-bore chest drain and under water seal drainage should be employed with thoracentesis in refracted cases.^8^

## Conclusion

Suspicion of primary pneumothorax should be heightened in apparently healthy babies who require minimal or no resuscitation at birth but develop sudden breathlessness. With prompt diagnosis and early administration of supplemental oxygen, conservative treatment may be successful with complete resolution.
